# Engaging scientists: An online survey exploring the experience of innovative biotechnological approaches to controlling vector-borne diseases

**DOI:** 10.1186/s13071-015-0996-x

**Published:** 2015-08-10

**Authors:** Christophe Boëte, Uli Beisel, Luísa Reis Castro, Nicolas Césard, R. Guy Reeves

**Affiliations:** UMR_D 190 Emergence des Pathologies Virales, Aix Marseille Université, IRD (Institut de Recherche pour le Développement), EHESP (Ecole des Hautes Etudes en Santé Publique), 27 Bd Jean Moulin, 13385 Marseille, Cedex 5 France; Culture and Technology in Africa, Faculty V: Cultural Studies, Universität Bayreuth, Bayreuth, Germany; History, Anthropology, and Science, Technology, and Society (HASTS) MIT, 77 Massachusetts Avenue, Cambridge, MA 02139 USA; CNRS-MNHN, UMR 7206 Eco-Anthropologie et Ethnobiologie, Dept Hommes, natures et sociétés, Musée de l′Homme 17 place Trocadéro 75016, Paris, France; Graduate School of Asian and African Area Studies, Kyoto University, Research Bldg. No. 2, Yoshida-Honmachi, Sakyo-ku, 606-8501 Kyoto Japan; Department of Evolutionary Genetics, Max Planck Institute for Evolutionary Biology August-Thienemannstrasse 2, 24306, Plön, Germany

**Keywords:** Biotechnology, Communication, Controversy, Disruptive technologies, Genetically modified organisms, Innovation, Mosquitoes, Public health, Public, Scientists, Technology, Perception, Transgenic, Wolbachia

## Abstract

**Background:**

Pioneering technologies (e.g., nanotechnology, synthetic biology or climate engineering) are often associated with potential new risks and uncertainties that can become sources of controversy. The communication of information during their development and open exchanges between stakeholders is generally considered a key issue in their acceptance. While the attitudes of the public to novel technologies have been widely considered there has been relatively little investigation of the perceptions and awareness of scientists working on human or animal diseases transmitted by arthropods.

**Methods:**

Consequently, we conducted a global survey on 1889 scientists working on aspects of vector-borne diseases, exploring, under the light of a variety of demographic and professional factors, their knowledge and awareness of an emerging biotechnology that has the potential to revolutionize the control of pest insect populations.

**Results:**

Despite extensive media coverage of key developments (including releases of manipulated mosquitoes into human communities) this has in only one instance resulted in scientist awareness exceeding 50 % on a national or regional scale. We document that awareness of pioneering releases significantly relied on private communication sources that were not equally accessible to scientists from countries with endemic vector-borne diseases (dengue and malaria). In addition, we provide quantitative analysis of the perceptions and knowledge of specific biotechnological approaches to controlling vector-borne disease, which are likely to impact the way in which scientists around the world engage in the debate about their value.

**Conclusions:**

Our results indicate that there is scope to strengthen already effective methods of communication, in addition to a strong demand by scientists (expressed by 79.9 % of respondents) to develop new, creative modes of public engagement.

**Electronic supplementary material:**

The online version of this article (doi:10.1186/s13071-015-0996-x) contains supplementary material, which is available to authorized users.

## Background

Over the last 100 years there have been numerous dramatic successes in the control of devastating human diseases vectored by insects, this includes the stable elimination of malaria from almost 100 countries [1] and the global control of urban yellow fever epidemics [2]. These successes have been brought about by a variety of technologies that at their inception were considered to be both experimental and pioneering. However, once the relative effectiveness of a vector-control technique becomes widely established their state-of-the-art status is often forgotten and in time becomes an obscure historical footnote [3]. Currently, in the context of inadequate regional control of some vectored diseases (e.g. malaria in sub-Saharan Africa) and newly emerging diseases (e.g. dengue), there has been an increasing interest in an innovative class of insect control approaches which use modern biotechnological techniques as a means to limit transmission [4, 5]. This has already resulted in large-scale open field trials of two biotechnological techniques in Australia, Indonesia, Vietnam, Cayman Islands (U.K.) and Brazil with a species of mosquito that is responsible for transmitting dengue to humans around the globe (*Aedes aegypti,* [6–10]). The first of these experimental approaches seeks to establish maternally inherited endosymbiotic bacteria at high frequency in target *Aedes aegypti* populations as a means to reduce their capacity to vector a range of diseases. This anti-pathogen tech nique was field trialed for the very first time in the Australian city of Cairns in 2011 and was largely developed by researchers at Australian universities [8, 9]. The second biotechnological technique uses a recombinant genetic engineering approach to render released males partially sterile as a means to reduce the size of target *Aedes aegypti* populations. This technique was largely developed by a commercial company (Oxitec Ltd. based in the United Kingdom) and was first field trialed in 2009 in the Caribbean Cayman Islands [7]. This was only the second time a transgenic insect had ever been intentionally released and was the first release of a transgenic mosquito (the earlier series of trials had released a transgenic moth pest of cotton in the USA, [11, 12]). Both the transgenic and the *Wolbachia* approaches require the release of large numbers of manipulated mosquitoes into human communities. These techniques, and other related biotechnological approaches have been argued as having the potential to positively revolutionize the control of diseases vectored by insects to humans, plants and animals [4, 5, 13–15]. In common with many other innovative technologies, if any of these diseases control techniques prove to be attractive alternatives to currently available methods this will inevitably result in the disruption of established approaches. Furthermore, the novelty of aspects of these experimental techniques has the potential to sustain and amplify controversy both in the general public and also among scientists [4, 16–21]. Consequently, engagement with stakeholders was repeatedly highlighted as of critical importance during the very early consideration of experimental trials of all these biotechnological approaches [4, 14, 16, 17, 22–29]. While there continues to be an understandable focus on the perceptions of the general public [16, 20, 22, 25, 30–32] there has been less work on how scientists in diverse disciplines and backgrounds perceive these novel biotechnological techniques (though see [17, 33–38]).

Herein, we examine the opinions and awareness of scientists on the topic of innovative biotechnological techniques by conducting an invited written survey of 1889 individuals expert in various aspects of diseases vectored to humans by arthropods. We show that despite extensive media attention on global and regional scales (e.g. [18, 21, 39–41]); awareness of key developments is consistently low. This is despite the fact that large numbers of the very scientists most likely to be impacted by developments were included in the survey (e.g. 389 of the sampled scientists spent more than 50 % of their time working in disease-endemic countries and report a substantial focus on applied research). However, in only one instance are we able to establish that awareness of pioneering developments exceeded 50 % on a national or regional scale.

The survey respondents include a sizeable sample from non-disease-endemic countries (where all these biotechnology techniques were initially developed) and the large communities of researchers in disease-endemic countries (where these approaches may eventually be applied). Crucially, our sample includes a large number (75 %) of disinterested scientists not directly involved in the development of these biotechnological approaches, but still expert in some aspect of vector-borne diseases. By contrasting these and other partitions of respondents’ backgrounds it was possible to identify factors that appear to strongly influence their views towards these pioneering techniques. We find that factors including their scientific expertise, citizenship, and their degree of professional involvement in biotechnology all have profound impacts on: (1) how and when they are first informed about pioneering and experimental techniques, (2) their attitude towards public engagement, and (3) their perceptions of innovative technological approaches. Given the limited capacity to communicate on global or continental scales demonstrated below, even with experts in fields potentially directly impacted by this technology, we argue that regional or nationally targeted engagement strategies represent the most promising approach to increasing the dissemination of information. Furthermore, increasing the awareness of key developments in the well established large pool of global scientists already expert in the general topic of vectored diseases could be an important step in enhancing local scientists’ role as contacts for decision makers and the public concerning rapidly developing innovative technologies.

## Methods

The e-mail addresses of corresponding authors were harvested from the web-of-science (Thomson Reuters) database using a series of keyword searches (Additional file 1) to identify active researchers publishing in the period 2005–2012 likely to be expert in at least one aspect of human or animal vectored disease. This list of e-mails was further parsed to yield 14747 unique addresses (Additional file 1). An invitation to participate in the survey was sent 4th October 2012 with the subject heading: Survey Invitation '*Transgenics and Vector-borne Diseases*' (Additional file 2). Further, 5 follow-up reminders were sent until the 11^th^ December 2012 (Additional file 2). The survey was conducted using the QuestionPro web-based platform (QuestionPro.com). The full text of the survey is provided in the Additional file 3. Throughout the text relevant questions are identified from the Additional file 3 by their respective number prefixed by the letter ‘Q’. Data exploration and statistical analysis was performed using JMP (SAS Institute) and Filemaker Pro (Filemaker Inc.). In total there were 1889 completed surveys from respondents indicating that their work was *‘somehow related to human or animal diseases that are transmitted by arthropods’* (Q1). Only 27 substantially incomplete surveys were discarded from analysis. The questionnaire was viewed 4046 times and started by 2771 responders. Most of the scientists working on vector-borne disease reported (Q2, Additional file 3) activities on: malaria 862 (45.6 %), dengue 778 (41.7 %), tick-borne diseases 377 (20 %), chikungunya 341(18.1 %), filaria 205 (10.9 %), Japanese encephalitis 191 (10.1 %), Chagas disease 317 (9 %), entomopathogenic fungi 70 (3.7 %), and microsporidia 27 (1.4 %). Among them 1245 (65.9 %) reported working on malaria or dengue, while 405 (21.4 %) declared activities on both diseases. To assess the extent to which the research interests of respondents reflected the output of the whole field of vector research during the relevant time period, title keyword searches were performed using the web-of-science database (Additional file 4). The correlation between this crude proxy for the research output of the whole field and that reported by the survey respondents was high, 0.91 for 2012 and 0.87 for the combined period 2005–2012, indicating a highly representative sampling from this perspective. Broadly speaking, all the diverse topics were representatively sampled with only malaria being somewhat underrepresented (though given that a prior smaller survey [33] specifically examined malaria researchers perceptions of transgenic mosquitoes the more extensive scope of the current data set complements the earlier study). Among the respondents, 720 (38.1 %) were citizens of a country endemic (Q3, Additional file 3) for either malaria or dengue as defined by the WHO and CDC in (2013, healthmap.org). Most respondents were resident in countries they were citizens of, with only 287 (15.2 %) being resident aliens (Q4, Additional file 3). Finally, a majority 1411 (75 %) of the respondent’s research was unrelated to transgenesis (including paratransgenesis, Q12, Additional file 3), with only 478 (25 %) reporting any involvement in transgenic research. The substantial sample size of both these groups permitted perceptual differences and similarities between these groups to be robustly explored. Respondents selecting ‘*No opinion’* or failing to select any of the given options are excluded from the analysis of the relevant question.

## Results and discussion

The large and diverse dataset of 1889 respondents to the 30 questions (see Additional file 3 for details) was explored, focusing on 3 major questions relevant to the implementation of biotechnological vector control: (1) *How aware are scientists of novel scientific techniques and how do they obtain information about pioneering developments?* (2) *What are scientists’ attitudes towards public communication and how might that impact the role they play in engaging the communities in which they live?* (3) *Should experts with similar professional specialization be viewed as homogenous from the perspective of how they view science and technology?* These questions in the general area of public and scientific engagement have been repeatedly highlighted as liable to be important to the successful development of biotechnological approaches to controlling vectored diseases approaches [4, 14, 16, 17, 22–29]. However, it is also likely to be highly relevant to a range of other innovative technologies e.g., nanotechnology [42], synthetic biology [43, 44] or climate engineering [45]. The particular importance of the above questions to the control of vector-borne diseases partly stems from the fact that biotechnological strategies (particularly those utilizing recombinant DNA methods) in public health are associated with new perceived risks and uncertainties, possibly at a higher level than other currently employed disease control techniques (vaccines, bed-nets, prophylactic-drugs or indoor insecticide spraying). This is probably also because most of these novel biotechnologies require the long-term maintenance of large numbers of manipulated insects dispersing by flight throughout human communities (in some instances transgenics). Undoubtedly, the recurrent controversy about the use of genetically modified crops in agriculture is also a source of defiance towards extending genetic engineering to blood-feeding insects living in human communities.

Obviously complex ethical, democratic and presentational challenges arise, many of which have been considered in the context of public perceptions and rights ([4, 14, 16, 17, 22–29, 46] though see [47, 48]). However, this study focuses on the potentially more tractable question of how scientists communicate with other scientists about pioneering techniques and their perceptions of them. The importance of genuinely bi-directional exchange between the public and scientists is widely recognized as crucial for the democratic legitimacy of scientific research. Sociological research has also demonstrated the role this dialogue plays in the acceptance or rejection of scientifically developed solutions and for public trust in science [49–51]. While this two-way engagement presumes a clear-cut distinction between expert ‘scientists’ and the less well informed ‘public’ [52], the boundaries between these categories appear from the survey results to be more inexact than might be anticipated.

### How aware are scientists of novel techniques and how do they obtain information about pioneering developments?

**Fig. 1:**
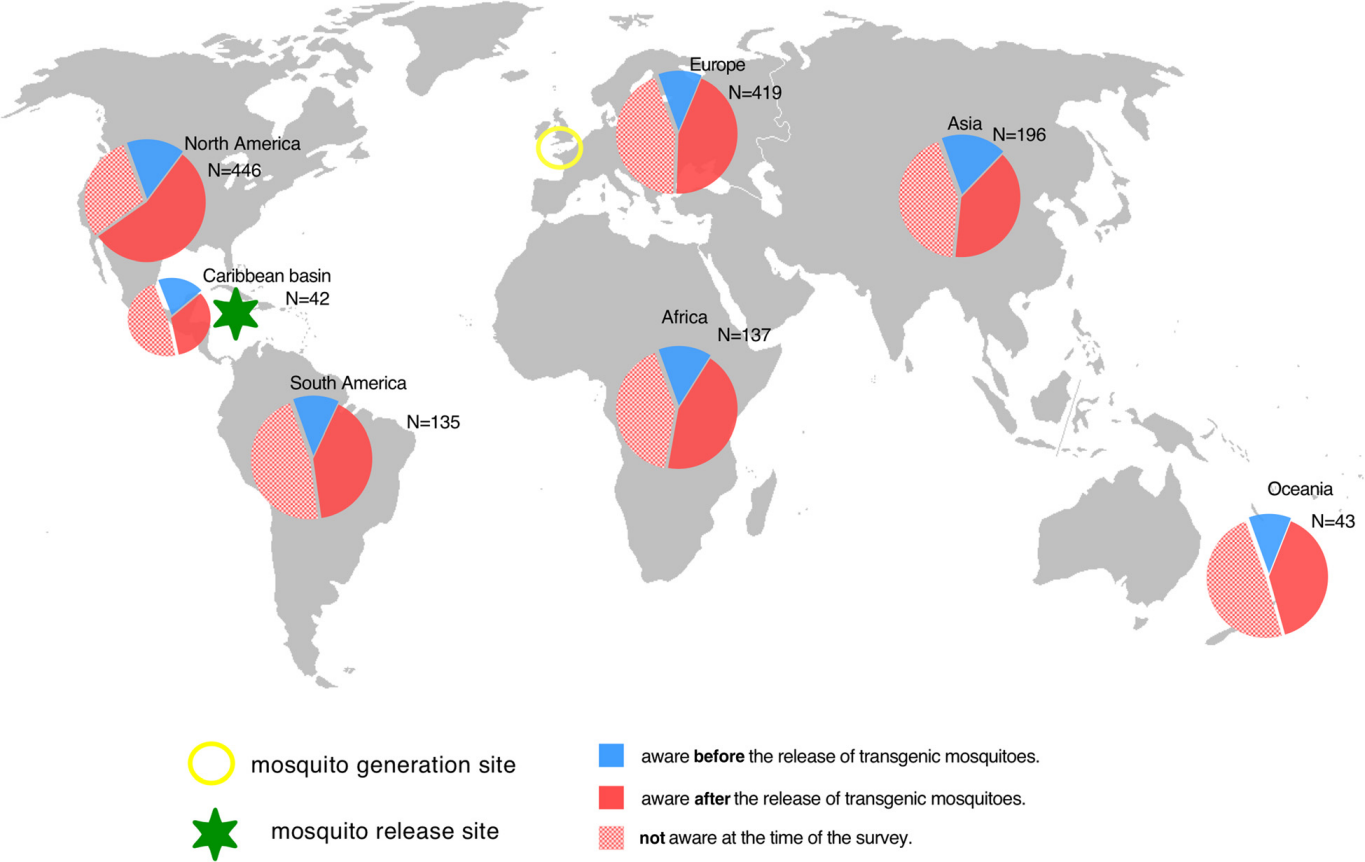
Regional and national awareness of the 2009 release of transgenic mosquitoes in the Cayman Islands before and after they occurred (Q20). Pie charts represent when respondents recall learning of the 2009 release of transgenic mosquitoes in the Cayman Islands

**Fig. 2:**
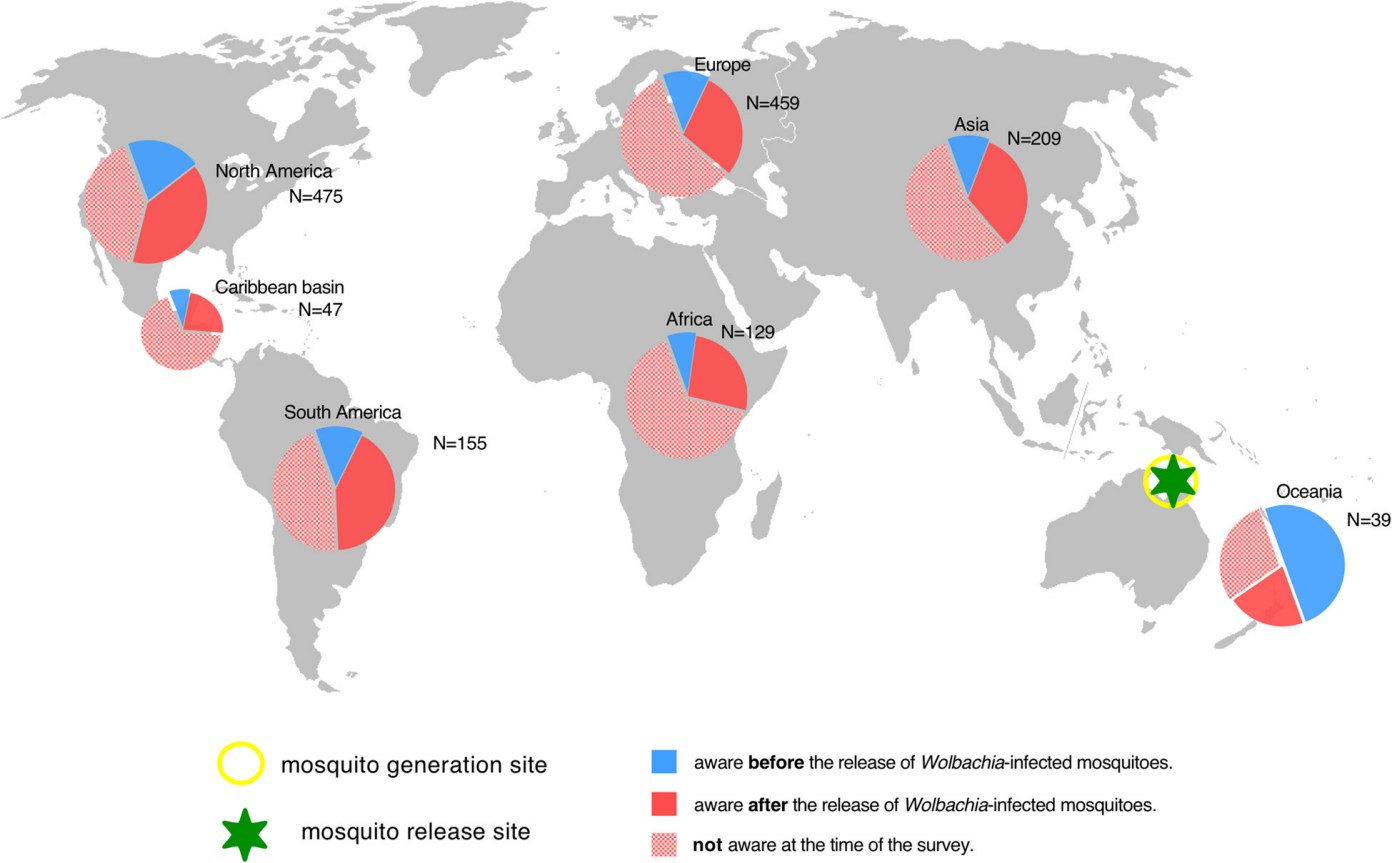
Regional and national awareness of the 2011 release of *Wolbachia*-transfected mosquitoes in Australia before and after they occurred (Q22). Pie charts represent when respondents recall learning of the 2011 release of *Wolbachia*-transfected mosquitoes in Australia

**Fig. 3:**
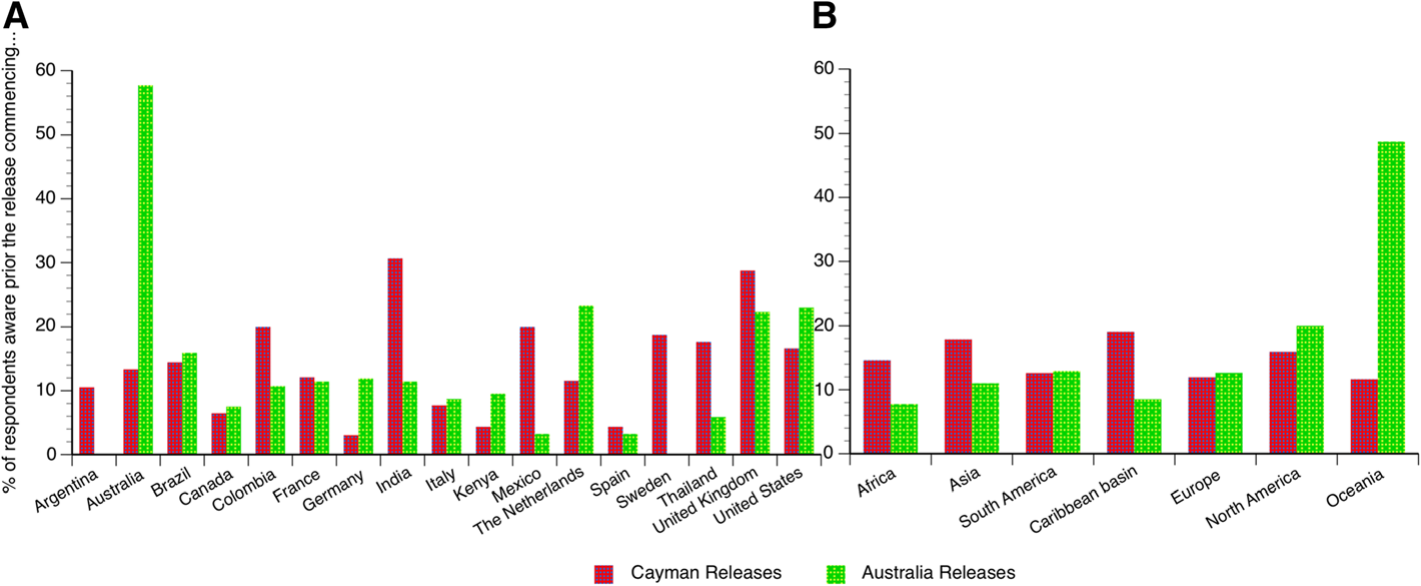
Regional and national awareness of pioneering mosquito releases before they occured. Awareness of either pioneering trial before releases occurred was uniformly low among expert scientists (<35 %) at national (3**a**) and regional (3**b**) levels. The only exception is the high national awareness achieved in Australia for the 2011 release that occurred in Queensland (Australia). The high awareness in the Oceania region for the Australian release (3**b**) should be viewed with caution as 66 % (Q20) and 72 % (Q22) of respondents from this region are Australian. Sample sizes are shown above the respective bars in (3**a**) and (3**b**). Individuals that were not able to remember when they learnt about the release or that where directly involved in it (Q21 and Q23) are excluded from the corresponding figure

To explore the awareness of scientists to pioneering developments, we asked respondents to recall when and how they became aware of the first field trials of two distinct approaches to control dengue fever. The first was the pioneering release of transgenic mosquitoes in 2009 in East End, Cayman Islands (an overseas U.K. territory in the Caribbean, [7]). The second was a release in 2011 of mosquitoes transfected with a maternally inherited endosymbiotic bacteria (*Wolbachia pipentis*) in Cairns, Australia ([8] – note that the date of release was incorrectly given as October 2011 rather than January 2011 in the survey text, see Additional file 3, Q22 for details and explanations concerning the validity of the analysis. From the responses given (Q20-24, Additional file 3) the most striking observation is the consistently small percentage of scientists (considering only the ones not directly involved in the releases) that were aware of either release before it started, consistently <35 % throughout the globe (Figs. 1, 2 and 3). The only exception is the Oceania region where pre-release awareness exceeded 50 % for the 2011 Australia release; however, this observation is largely driven by a high degree of awareness in Australia (Fig. 3a). In summary, with respect to either of these two pioneering trials, based on our sampling, there is only compelling evidence for a single nationally successful communication strategy and none for any globally successful ones.

**Fig. 4:**
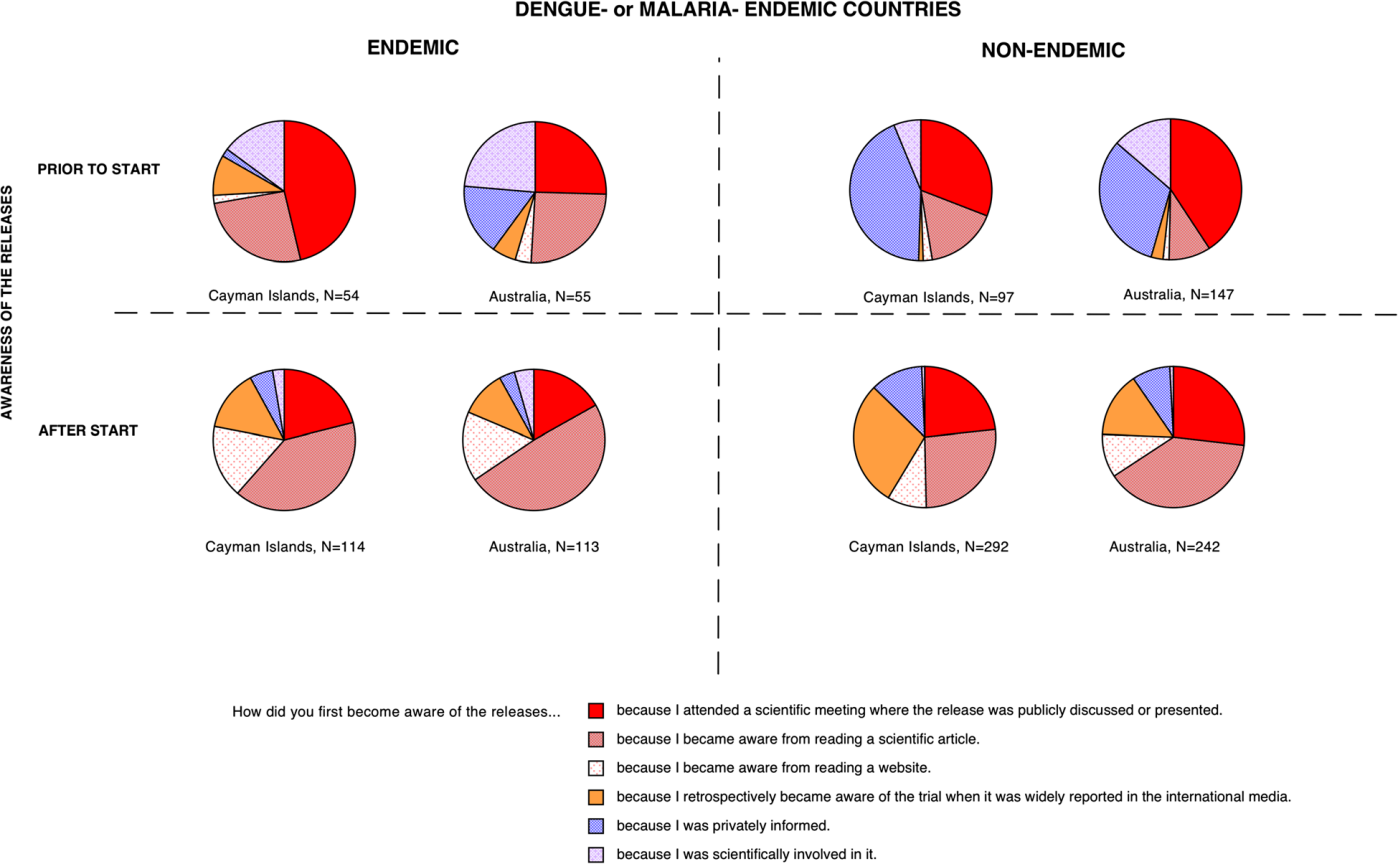
Information sources utilized by scientists for pioneering releases of mosquitoes in 2009 in the Cayman Islands and in 2011 in Australia (Q21 and Q23). In the small group of respondents aware of releases prior to their commencement (upper row of pie charts), a relatively high proportion of individuals from disease non-endemic countries (right columns) had access to private information that was unavailable to scientists from disease-endemic countries. Subsequent to releases, starting information sources utilized by scientists around the globe was fairly uniform. See note in Text S3 about the impact of the start date of the Australia trial being incorrectly given in Q22 as Oct 2011 rather than Jan 2011

Surprisingly, a large percentage of scientists were unaware of either release at the time they participated in the survey (>30-50 % in all regions, Fig. 1). This was somewhat unanticipated, as both releases had attracted a significant degree of global coverage in the general media [41, 53–56] and scientific press [18, 19, 21, 39, 57]. However, our survey suggests that this media attention did not translate into a high degree of global awareness among scientists despite 3 years having elapsed since the Cayman releases and 1 year since the Australia releases. Examining how experts recall first learning about these pioneering field releases we found a clear distinction in the information sources utilized by the minority of respondents aware before releases start compared to those who became aware afterwards (Fig. 4). For the majority of scientists that became aware after releases had commenced there is a remarkable consistency in the sources of information utilized, for both the Cayman and Australia releases. In all instances, conventional scientific sources such as scientific meetings and articles appear to play a predominant role (only the Cayman release appears to differ somewhat, probably due to increased media coverage of the associated controversy about the manner of the release, [18, 19, 21, 41]). With respect to this high reliance on conventional scientific sources, it is notable that academic institutions were associated with high levels of trust, e.g., 69.3 % Universities and 70.8 %, the WHO compared to 22.7 % for the media and 28.7 % for private business (Q25, see also Additional file 5).

Interestingly, for the minority of scientists that were aware of either release before they commenced (<35 %), it is clear that being privately informed is an important route of information among scientists from non-endemic countries (Fig. 4). However, these networks of private communication do not appear to extend to the same extent to scientists from disease-endemic countries (Fig. 4). Finally, we observe that the widespread lack of awareness of key developments illustrated in Fig. 4, also extended to contemporaneous events in 2012. Respondents were asked to state if they were aware of any current open releases of transgenic mosquitoes (Q26). Throughout the period of the survey in 2012 releases were occurring in the Brazilian city of Juazeiro (Bahia). However, only 29.1 % (N = 550) correctly responded ‘yes’ to this question, though among researchers involved in transgenic research (Q13) this was somewhat higher at 43.3 % (N = 207). Interestingly, Brazilian scientists were among the most informed with 55.9 % (N = 87 of 152, Q26) reporting being aware of the releases; however, only 42.5 % (N = 37of 87, Q27) were right about the releases happening in their own country. Though the Brazilian release (starting in 2011) cannot be considered pioneering in the same way as the Cayman 2009 or Australia 2011 releases, the fact that awareness is highest in the country where the trial is conducted is consistent with the high national awareness of the 2011 *Wolbachia* release in Australia (Fig. 2). Unfortunately, the survey did not include any respondents resident in the Cayman Islands, so it was impossible to determine if high local awareness was also the case for the 2009 Cayman island release. Four hundred and seven respondents incorrectly identified at least one country other than Brazil as a release location in 2012 (Q27), these included Malaysia (N = 120), Cayman Islands (N = 34), Australia (N = 96), USA (N = 51), Thailand (N = 27), Mexico (N = 27), and various African countries (N = 45). Why some of these locations were repeatedly identified can in some instances be reasonably be speculated upon. For example, Malaysia and the Cayman Islands were the sites of earlier releases (2009–2011) of transgenic mosquitoes that had ceased prior to the 2012 survey. The USA reports are likely to be related to a pending application to release transgenic mosquitoes in the Florida Keys [39]. Similarly, the incorrect identification of Mexico as an open release site is likely related to contained outdoor caged experiments in the Mexican state of Chiapas [58]. Respondents identifying Australia as a release site (N = 96) probably mistook the *Wolbachia* releases in 2011 for a transgenic technique [8]. Concerning the 45 respondents identifying African countries as release locations, it is less clear what might be the basis, as to our knowledge no such releases of manipulated mosquitos had been officially proposed (though see [59]). It is possible that this incorrect identification might be related to an existing project with conventional sterile insect technique releases of mosquitoes in Sudan [60]. Interestingly, of the 45 respondents indicating an African release of transgenic mosquitos in 2012 only 8 were citizens of African countries, indicating that this belief is not likely to be based on local information. Collectively, the widespread misunderstandings about what releases were genuinely occurring in 2012 (Q26 & Q27) and the low degree of awareness of two earlier pioneering releases (Fig. 1, 2, Q20 & Q22) emphasize the fact that even among the scientists basic knowledge can often be quite incomplete and imprecise.

### What are scientists’ attitudes towards public communication and how might that impact the role they play in engaging the communities in which they live?

**Fig. 5:**
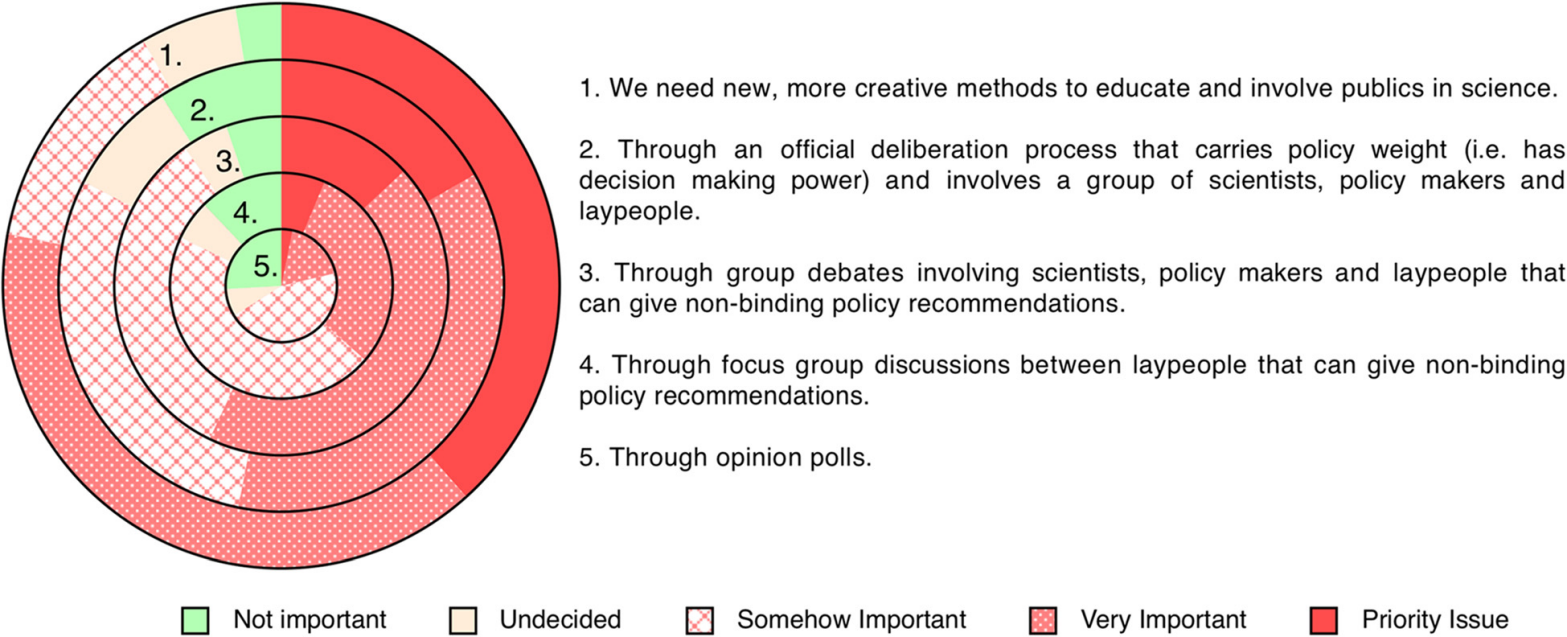
Scientists opinions on how the public could be involved in scientific decision making (Q19). Radial bar chart representing the five responses to the question ‘*How do you think citizens can be usefully involved?*’ There is a strong consensus for a need for new creative methods to involve the public in science (77.9 % selected ‘*very important*’ or ‘*priority issue*’). There is much less enthusiasm for the use of opinion polls (21.3 % selected ‘*very important*’ or ‘*priority issue*’)

Given the difficulties in achieving effective information dissemination in untargeted global communication strategies it is probable that for public engagement to be effective it will most likely need to rely, at least to some extent, on local scientists (independent of any increased democratic legitimacy it generates). Consequently, we were interested to explore what scientists’ professional involvement with the public looks like and how they value this activity. The survey reveals that only a very small proportion of scientists (5 %) communicate more than once a month with non-specialist audiences (Q17), while more than 80 % have very limited interactions or none at all (Additional file 6). This occurs despite 53.8 % of respondents fully agreeing with the value of communicating with the public about their work compared to only 11 % who expressed no value in doing so (Q15, Additional file 6). When asked how science communication with the public could be improved, 77.9 % of the respondents indicated the need for more creative methods to educate and involve the public in science (Q19, Fig. 5). This indicates that most experts in vectored diseases consider the existing opportunities to communicate with a non-scientific audience as inadequate to the task.

**Fig. 6:**
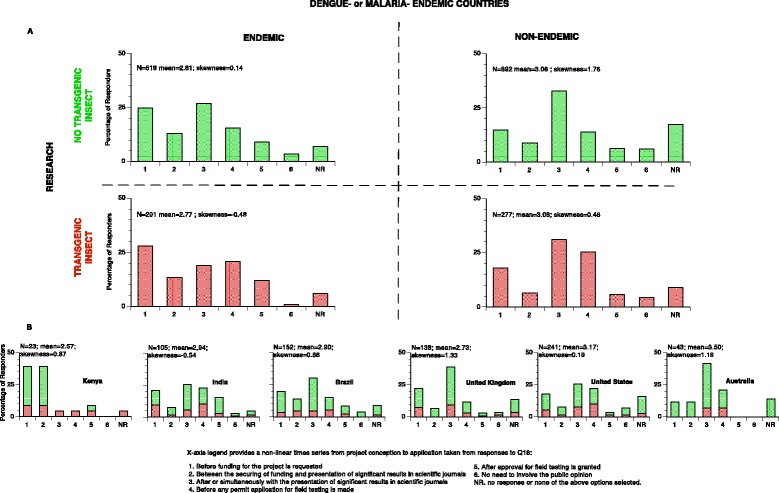
Scientists preferences for the earliest stage of public consultation in scientific projects (Q18). Respondents were asked *‘When do you think citizens should become involved?*’. Multiple responses were possible, but only the earliest is represented here. Histograms **a**, are grouped by whether respondents are a citizen of a malaria or dengue endemic country (Q3) and if their field of research (Q12) includes genetically modified insects (red) or not (green). **b** Country specific histograms from nations with the largest number of respondents per continent. Field of research is colored as above. X-axis of all histograms provides a non-linear time series from project conception to deployment. ‘NR’ indicates that none of the provided responses were selected

In keeping with the general desire of scientists to communicate with the public, almost all scientific articles and reports on novel biotechnological approaches included statements about the importance of public engagement [4, 14, 16, 17, 22–29]. However, despite this apparent consensus there is potentially a very broad range of understandings of what might actually constitute meaningful public engagement [26]. In order to address this question we asked respondents to provide their opinion on the specific question of *when* they thought the public should be involved in projects (Q18). Five response options ranging from early stages ‘*before funding is requested’*, to very late stages ‘*after approval for field testing is granted’* as well as ‘*no need to involve the public opinion*’ were provided and multiple responses were possible (Additional file 3). The results are presented in Fig. 6a as the earliest time point of public engagement indicated by each respondent. Given the broad scope of the response options provided, it is perhaps unsurprising there is a wide range of opinions on the fundamentally important question of at what stage public engagement should be initiated. There is however a strong consensus 86 % (N = 1660) that public engagement should commence ‘*Before any permit application for field testing is made*’. More generally, it is striking that responses do not approximate uni-modal distributions with a sizable number of respondents opting for early engagement (‘*before funding is requested’*) with another larger group opting for initiation at later stages (‘*After or simultaneously with the presentation of significant results in scientific journals.*’ or ‘*Before any permit application for field testing is made’*). While the four divisions shown in Fig. 6a have similar means (2.77-3.08) they exhibit varying degrees of skew towards or away from early engagement; most striking is that scientists from disease-endemic countries selected the earliest response ‘1’ (‘*before funding is requested’*) more frequently than did scientists from non-endemic countries (25 % and 28 % versus 15 % and 18 %). Figure 6b also shows large disparities are clearly discernable at national levels, particularly with respects to the frequency of response ‘1’. For example 39 % of Kenyan respondents considered that the public should be involved ‘*before funding is requested*’, while in Australia and the USA the frequency was 12 % and 18 % respectively. Clearly within such a large dataset there is considerable potential for regional and national variation; however, a quantitative awareness of such predispositions could prove valuable when planning research, collaborations or guidelines in different locations around the globe [24, 27, 29, 61].

### Should experts with similar professional specialization be viewed as homogenous from the perspective of how they view science and technology?

**Fig. 7:**
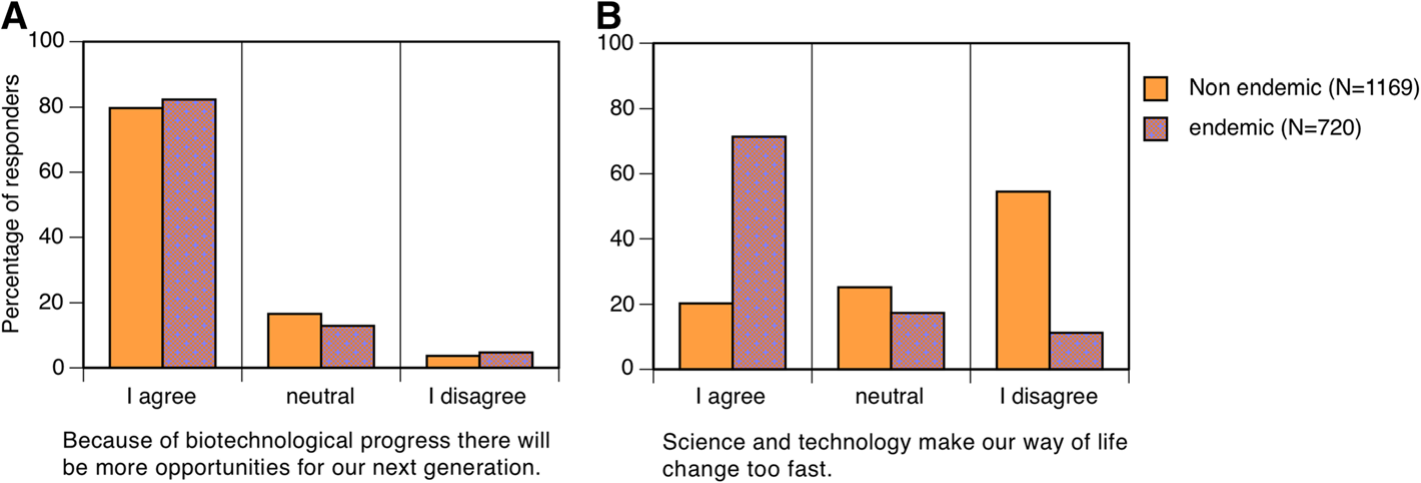
Perceptions of science and technology by scientists (Q24). Scientists were asked ‘*As a scientist but also as a citizen, how much do you agree or disagree with the following statements about science, technology and society?’*. The specific statements are reproduced below the histograms. There is a very high degree of agreement that biotechnology will provide new opportunities to future generations between scientists that are citizens of disease-endemic countries and those of non-endemic countries. However there is a very striking contrast between the same groups as to the perceived desirability of the pace of change due to science and technology

The large size of the dataset and its breadth in terms of the diversity of respondents permits the exploration of diversity among scientific experts with distinct disciplines and backgrounds. Possibly the most striking differences relate to general perceptions about technology and trust as scientists but also as citizens (Q24 & Q25, reproduced from [62]). While there are numerous similarities, there are also some striking differences. For example, there is strong agreement between scientists who are citizens of both endemic and non-endemic countries that biotechnological progress will result in increased opportunities Fig. 7a. However there is a striking contrast in their perceptions of whether science and technology make our lives change too fast, with a 71 % of endemic scientists agreeing and only 20 % of non-endemic scientists (Fig. 7b). While these observations do not provide direct insight into how transnational collaboration should be conducted they do illustrate the potential for fundamentally different viewpoints existing even among expert scientists. Furthermore, it appears to reflect that professional expertise as scientists does not divorce them from the influence of the societies they live in. In other words, scientists are also and in many circumstances, a public for other scientists.

The overall aim of this survey was to elucidate scientists’ knowledge of and opinions on a currently emerging technology at an early stage in its public and scientific evaluation. This was done for the mass release of biotechnologically manipulated mosquitoes for the control of human diseases (e.g., dengue). Only 25 % of the scientists in the survey indicated any direct involvement in developing the technology, with the remaining 75 % being in related but distinct disciplines (many of which are likely to be impacted should any novel methods prove to be effective). Probably the most publically visible phase of technology development commenced for the two most advanced techniques in 2009 and 2011 with large-scale experimental releases in human communities. Overall the most striking result of the survey is the small percentage of scientists who were aware of either release before it started, consistently less than 35 % throughout the world (Fig. 1 and 2). This is despite a strong consensus among respondents 86 % (N = 1660) that engagement should commence ‘*Before any permit application for field testing is made’*.

If experts working in the scientific field of vector control were not reached by any means of communication prior to high-profile pioneering trials commencing we can reasonably ask who then was reached at all? As the intense public controversy around genetically modified food shows, such a lack of engagement and inadequate dissemination of accurate information can backfire at the point of widespread implementation of new technologies [49, 63]. Equally, this lack of information calls into question the ethics and the democratic legitimacy of such research: if even scientific colleagues are not informed, how then do potentially disagreeing parties have a chance to voice their concerns? Furthermore, the widespread misunderstandings about what releases were genuinely occurring contemporaneously at the time of the survey (Q26 & Q27) and the low degree of awareness of two earlier pioneering releases (Figs. 1, 2 and 3, Q20 & Q22) emphasize the fact that even among experts basic knowledge is often incomplete and imprecise. This clearly highlights significant gaps in the communication processes occurring within the scientific community. Of the scientists from disease non-endemic countries that were informed about releases prior to them occurring 29 %-41 % obtain information via private sources (Fig. 2). However, these private sources were not equally available to scientists in disease-endemic countries where only 4 %-6 % accessed them (Fig. 4). This result points towards continuing inequalities in information sharing between scientists from the so-called developing and developed countries. Generally, the survey points to a need to improve communication between peers (Figs. 1 and 2), but more specifically to extend existing communication sources (including informal ones) to more fully include experts in disease-endemic countries where this technology is most likely to be used.

The survey showed that only scientists in the country where a release took place were widely aware of developments (Australia, Figs. 1 and 2 and Brazil Q27). This underlines the effectiveness of communication at local and national levels in disseminating accurate information in a timely manner. With respect to designing and implementing future inclusive communication strategies the success of the Australian ‘*Eliminate Dengue’* project to achieve 70 % awareness prior to an up coming development (Fig. 2) is a notable achievement. Otherwise, the survey results indicate the aim of raising awareness even in groups of interested scientists is an extremely challenging one using conventional communication approaches. The survey results do however indicate that in attempting to increase awareness among experts it may be productive to strengthen traditional scientific communication methods (scientific articles and meetings) rather than relying on general media exposure (Fig. 4). This having been said, there were two meetings specifically focused on the topic of transgenic mosquitoes in the 9 months preceding the 2009 Cayman releases [28, 64], however this does not appear to have translated into a high degree of awareness of the upcoming pioneering development (Fig. 4). Expanding private information networks – through scientific collaboration and exchanges for instance - to include scientists from endemic countries would also appear to be a valuable component in communicating with the countries where these techniques are most likely to be experimentally tested or applied.

The survey also questioned scientists about their habits and opinions on communicating with the general public. The survey reveals that only a very small proportion of scientists (5 %) communicate more than once a month with non-specialist audiences (Q17), while more than 80 % have very limited interactions (Additional file 6). This finding is consistent with data reported for biomedical researchers were <75 % reported being in contact with journalists between 0–5 times a year [65]. While we did not directly explore the reasons for such a low level of engagement despite a high value being placed upon it (by 53.8 % of respondents), it is likely to require a multifaceted explanation. One probable factor is of course lack of time for a task some scientists might consider secondary to their research and professional duties. Equally, it could reflect a limitation in the access that many scientists have to effective means of communication--for example media-outlets may be perceived to be interested in only hearing from a small group of high-profile specialists [65]. Such explanations would need to be specifically investigated, but a better understanding could be valuable in helping lower the barriers between the majority of scientists enthusiastic about communication and the interested public. In the context of earlier studies detailing a correlation between the number of science communication activities and the perceived importance of public engagement [66, 67], it is interesting to note that there is a positive correlation in the current survey (Additional file 7) between the frequency at which scientists report communicating with the public (Q17) and a preference for earlier engagement of the public in scientific projects (Q18).

However, regardless of this correlation, a remarkable 77.9 % of the surveyed scientists indicated the need for more creative methods to educate and involve the public in science (Q19, Fig. 5). This shows that most scientists consider the existing opportunities to communicate with non-scientific audiences as inadequate. The pervasive desire for new and creative communication methods by vector scientists is thus a major result of this survey. It suggests a need for more tailor-made and imaginative approaches to science communication [65], potentially motivating collaborations between scientists, professional communicators, artists and other professions to better use existing tools and to develop new ones ([68, 69] including online [36]). In the context of unconventional communication techniques, unexpectedly, our survey has proven to be the largest single tool in globally disseminating information about key developments in this field (see Fig. 1). While, the information conveyed may be of a superficial nature it is noteworthy that 80 % of respondents to a related survey of Nigeria scientists stated that participation would inspire them to seek additional information about transgenic mosquitoes [34].

In a broader sense the survey can be viewed as reflecting that scientists are in many situations themselves a 'public' of science. Just as laypersons’ competences and awareness of technological issues differ, it is often not reasonable to assume that ‘a scientist’ understands all relevant aspects- even in his or her own subfield of science [70]. In this sense a simple binary division between science and lay knowledge appears fraught. Instead we see a clear need to place scientists among their society and not outside of it [52]. More symmetrical communication and experimenting with creative ways of engaging may prove productive in bringing the sciences and their various publics into effective dialogue with each other.

### Limitations of survey

Surveying a diverse set of professionals dealing with vector-borne diseases and public health necessitates making choices in sampling. The method of selecting for corresponding authors of papers dealing with vector control through the Web of Science has already been used successfully in a previous survey [33]. Alternative ways of sampling could have been to restrict participation to authors that have published a minimal number of papers [71], or only consider authors of highly cited papers [72, 73]. The underlying assumption would be that a wider inclusion might have biased the survey by including authors whose work on vector control is only a peripheral part of their activity. However, as we chose to include only corresponding authors this bias should be limited. In addition, our wider sampling method has the advantage to get young scientists involved in the survey instead of favoring a narrow selection of senior scientists. This also avoids the risk of an overrepresentation of domains where publication production is higher. Additionally, we were explicitly keen to involve the wider scientific community concerned with vector control, rather than a narrow selection, which tends to be biased towards a one author, one voice system. Wider sampling, we suggest, offers us the possibility to access the opinions and experiences of a “community of practice” [74], namely the scientists who share a profession and a common concern in their engagement in vector control. We believe this is important as it does not restrict us to include only scientists working in transgenic vector control means, but allow us to include a representative sample of scientists working on other technologies and approaches of vector control (see also Additional file 4).

Information about science is only one factor in creating conditions for a democratic engagement with science and scientific results. Accepting and trusting results and biomedical interventions rather requires broad reflect on the normative commitments of science, as well as its economic and institutional distributions and politics [49, 50, 70, 75, 76]. However, information about science and public trials of scientific interventions is nevertheless to be seen as one of the prerequisites for such broader deliberations to take place. In this sense, the present study focuses on one very limited aspect of the public understanding and engagement with science, but nevertheless a crucial one we suggest.

## Conclusions

Questioning the general public about their knowledge, perception and fears concerning novelties and innovations is a well established approach in social science studies [49, 50, 70, 75]. While communication approaches that aim at rectifying perceived deficiencies or gaps in knowledge have repeatedly been shown to be largely ineffective for the general public, as far as we are aware it remains to be determined how useful they might be for experts [77]. Here we took a converse approach by examining the perception and awareness of scientists to a specific emerging technology.

This revealed a lack of communication among a substantial proportion of the scientific community and that some of the information that is circulating can be inaccurate. This finding was common to the 74.7 % of scientists in the study who were not directly involved in the development of the technology, but also to the 26.3 % that reported being actively involved. Our work also suggests that scientists should be seen as publics in the societies they live in and that a large majority of them would value more extensive public engagement, with 79.9 % in favor of finding more creative methods to do this. Making extensive connections between specialists involved in technology development and expert scientists in related fields is clearly challenging. Initiatives such as biohacker spaces [78] might be one of the many ways to open a space of mutual engagement, including with the general public.

In summary our results strongly support the value of prioritizing mutual knowledge building between specialists scientists involved in technology development and scientists in related fields as a means to promote a genuinely two-way dialogue. Furthermore, because technology specialists constitute only a very small proportion of the scientific community, engaging with numerically much larger pools of experts that are already locally established has numerous practical advantages (this is in addition to affording an increased legitimacy to decision making processes by facilitating prominent roles for local scientists). Clearly, an inclusive dialogue between scientists can only facilitate its expansion to include the general public. While our study focused on applications and early developments in a specific biotechnology, most of our conclusions are likely to have wide applicability to numerous diverse fields such as nanotechnology, synthetic biology or climate engineering.

## Additional files

Additional file 1:
**Keywords used to identify researchers working in the field of vector-borne diseases.**
Additional file 2:
**Invitation and reminders sent to participate in the survey.**
Additional file 3:
**Full text of the survey and notes.**
Additional file 4:
**Representativeness of survey respondents’ field or research (Q2) relative to the publication output of field (based on Web-Of-Science database).**
Additional file 5:
**Level of trust in sources of information about the balance between risks and benefits concerning the release of transgenic mosquitoes (Q25).**
Additional file 6:
**Reported frequency of communicating and discussing science with a non-specialist audience in events (science fairs, TV/Radio shows, popular press….) not including teaching at a university or at a school (A) (Q17) and its perceived value (B) (Q15).**
Additional file 7:
**Correspondence analysis between frequency of communication (Q17) and attitudes to early engagement of the public in the research process (Q18).** Red crosses represent the level of implication of scientists in communicating and discussing science with a non-specialist audience. Blue squares indicate how early the engagement of the public in the research process is expected by researchers (the lower the number is, the earlier the engagement is).
