# Supplementary material for: Engaging scientists: An online survey exploring the experience of innovative biotechnological approaches to controlling vector-borne diseases
Source: Parasit Vectors. 2015 Aug 10;8:414. doi: 10.1186/s13071-015-0996-x (PMC4530488; doi:10.1186/s13071-015-0996-x)
Supplement: Additional file 1: — Keywords used to identify researchers working in the field of vector-borne diseases. [file 13071_2015_996_MOESM1_ESM.pdf]

Additional File 1. Questionnaire used for the survey

Note :

(*)The start data of the Australian release was incorrectly given in Q22 as October 2011 rather than the correct data of January 2011.
Rather than omitting entirely data from the Australian trail (Q22 and Q23) and presenting only data for the Cayman releases (Q20 and Q21, where no such error was made) we have retained the Australian data for the following reasons.

(1) Only a small number of respondents that recall learning of the trial between Jan and Oct 2011 would have misclassified themselves as having known prior to releases occurring. This would have had the effect of artificially inflating the small group of people reporting they knew prior to the releases.
(4) There is a very high similarity in the groupings shown in Figure 2 between the 2 trials suggesting that any misclassification in one had a minimal impact.
(3) If misclassification in the 10-month in question period did commonly occur it would most likely be evenly distributed across the world meaning that the striking difference in awareness in Australia (Fig.1C) is unlikely to be explained by this.
(4) The small error only applies to the timing of the Australia trial (and not to its information sources utilized) and not the Cayman trial. Presenting the Cayman data in the absence of the Australian trial would fail to reflect for the reader that there is a general communication problem, rather than something uniformly unique to the Cayman trial.

We thank Luke Alphey for drawing our attention to this point.

Q1. Is your work somehow related to human or animal diseases that are transmitted by arthropods?

- Yes: %
- No: % (If the answer was no, then the survey was politely terminated)

Q2. Which vector-borne disease or vector parasite are you working on? (more than one answer is possible)

- Malaria
- Dengue
- Japanese Encephalitis
- Chikungunya
- Tick-borne diseases
- Other viral diseases
- Chagas disease
- Filaria
- Entomopathogenic fungi
- Microsporidia
- None of them, I work on arthropod only.
- Other

Q3. Which country(-ies) or territory(-ies) are you a citizen of?

Q4. In which country or territory are you resident most of the time?

Q5. What percentage of your time do you spend working in a dengue-endemic area?

Q6. What percentage of your time do you spend working in a malarious area?

Q7. In which area are you spending most of your work time?

- Africa
- Asia
- Central, South America and the Caribbean
- Europe
- North America
- Oceania

Q8. What percentage of your research is field-based (vs lab-based)?

Q9. Do you consider most of your research...

- more fundamental than applied
- more applied than fundamental
- equally balanced between applied and fundamental

Q10. What is your major research domain?

- Ethics in Health
- Research Capacity building and strengthening
- Drugs
- Health Systems research and socio-economic aspects of disease research and control
- Immunology and vaccines
- Epidemiology, case management and control
- Parasite biology
- Vector control and biology
- Virology
- Other (please name)

Q11 Do you think that your research has potential effective applications for the control of vector-borne diseases in the next 10 years?

- Yes: %
- No: %

Q12 Is your research at least partly related to the question of transgenic insects (including paratransgenesis)?

- Yes: %
- No: %

If answer to Q12 is YES, then Q13, otherwise go to Q15.

Q13 Rate the percentage of your work dedicated to research in relation to transgenic mosquitoes?

Q14 Please indicate the number of peer-review papers / book chapters you have published for each of the different aspects since 2005 ***Note:*** *If you have written only one paper but if it deals in an equal manner with several aspects, then tick all the answers that apply otherwise please select only the one corresponding to the major topic of the paper(s).*

- ✓ Any aspect related to GM mosquitoes.
  - None:
  - Between 1 and 5:
  - Between 6 and 10:
  - more than 10:
- ✓ Molecular aspects related to GM mosquitoes (creation of GM strain of mosquitoes, molecular mechanisms of malaria/ mosquito interactions).

- None:
- Between 1 and 5:
- Between 6 and 10:
- more than 10:

✓ Ecological and/or evolutionary issues related to the potential use of transgenic mosquitoes.
- None:
- Between 1 and 5:
- Between 6 and 10:
- more than 10:

✓ Social and/or Ethical issues related to the potential use of GM mosquitoes.
- None:
- Between 1 and 5:
- Between 6 and 10:
- more than 10:

✓ Other topics.
- None:
- Between 1 and 5:
- Between 6 and 10:
- more than 10:

Q15. What is your opinion about this statement "Interactions with the public about my research are desirable".
- I fully disagree.
- I somehow disagree.
- Undecided.
- I somehow agree.
- I fully agree.

Q16. Would you agree for your research to be evaluated by non-specialists?
- Yes: %
- No: %

Q17. Not including teaching at a university or a school are you involved in events (science fairs, TV / Radio shows, popular press….) aiming at communicating and discussing science with a non-specialist audience?
- Not at all
- Once or twice a year
- Quite often (every two months)
- Very often (more than once a month)

Q18. When do you think citizens should become involved? (Multiple answers possible)
- Before funding for the project is requested
- Between the securing of funding and presentation of significant results in scientific journals
- After or simultaneously with the presentation of significant results in scientific journals
- Before any permit application for field testing is made
- After approval for field testing is granted
- No need to involve the public opinion
- No Opinion
- Other (please explain)

Q19 How do you think citizens can be usefully involved?

- Through opinion polls
- Through focus group discusssions between laypeople that can give non-binding policy recomendations
- Through group debates involving scientists, policy makers and laypeople that can give non-binding policy recommendations
- Through an official deliberation process that carries policy weight (i.e. has decision making power) and involves a group of scientists, policy makers and laypeople
- We need new, more creative methods to educate and involve publics in science

Q20 When did you first become aware of the worlds first open release of GM mosquitoes which started in Oct 2009 in the Cayman Islands?

- I was aware of it prior to the release starting
- I became aware of it sometime after the release had started
- I don't remember when I learned about it
- I was not aware of this release

Q21 is not accessed if the answer to Q20 is 'I was not aware of this release'.

Q21 I first became aware of the worlds first open release of GM mosquitoes, which started in October 2009 in the Cayman Islands... I first became aware of the worlds first open release of GM mosquitoes, which started in October 2009 in the Cayman Islands...

- because I was scientifically involved in it.
- because I attended a scientific meeting where the release was publicly discussed or presented.
- because I retrospectively became aware of the trial when it was widely reported in the international media.
- because I became aware from reading a scientific article.
- because I became aware from reading a website.
- because I was privately informed.
- I don't remember when I learned about it.

Q22 When did you first become aware of the release of Wolbachia-infected mosquitoes (a non transgenic technology), which started in Oct 2011 in Australia? *

- I was aware of it prior to the release starting.
- I became aware of it sometime after the release had started.
- I don't remember when I learned about it.
- I was not aware of this release.

Q23 is not accessed if the answer to Q22 is 'I was not aware of this release'.

Q23 I first became aware of the release of Wolbachia-infected mosquitoes (a non-transgenic technology) which started in october 2011 in Australia,

- because I was scientifically involved in it.
- because I attended a scientific meeting where the release was publicly discussed or presented.
- because I retrospectively became aware of the trial when it was widely reported in the international media.
- because I became aware from reading a scientific article.

- because I became aware from reading a website.
- because I was privately informed.
- I don't remember when I learned about it.

Q24 As a scientist but also as a citizen, how much do you agree or disagree with the following statements about science, technology and society?

	I disagree	I agree	neutral
Because of biotechnological progress there will be more opportunities for the next generation.			
Science and technology make our way of life change too fast.			
Research results from scientists employed at universities are more reliable than those from scientists employed by private businesses			

Q25 Concerning the balance between risks and benefits concerning the release of transgenic mosquitoes, how much do you trust the informations given by the following institutions?

- Private business
- Universities
- The World Health Organization
- Non Governmental Organizations
- Your National Parliament
- Your national biotechnology regulators
- The Media

Q26 Are you aware of any current open release of transgenic mosquitoes?
- Yes
- No

Q27 What location(s) are you aware of ongoing release(s)of transgenic mosquitoes?
Q28 What is your gender?
Q29 What is your date of birth?
Q30 How many years have you been working in research?
