# Supplementary material for: Engaging scientists: An online survey exploring the experience of innovative biotechnological approaches to controlling vector-borne diseases
Source: Parasit Vectors. 2015 Aug 10;8:414. doi: 10.1186/s13071-015-0996-x (PMC4530488; doi:10.1186/s13071-015-0996-x)
Supplement: Additional file 2: — Invitation and reminders sent to participate in the survey. [file 13071_2015_996_MOESM2_ESM.pdf]

Additional File 1. List of keywords used to identify researchers working in the field of vector-borne diseases.

In order to identify active researchers publishing in the field of vector-borne diseases between 2005 and 2012, we have been searching for corresponding authors in the Web of Science database using various combined queries: The search has been performed between 01-01-2005 and the 15th of September 2012 and it can be compiled as follow:

TS= (Anophel* OR
Culex OR
Aed* OR
mosquit* OR
Culici* OR
medic* entomolog* OR
veterinar* entomolog*)

OR

TS= (vir* AND mosquit*) OR
(malari* AND mosquit*) OR
(vector-borne AND diseas*) OR
(arthropod-borne AND diseas*) OR
(microsporidi* AND mosquit*) OR
(GM insect AND human health) OR
(GM insect AND animal health) OR
(GM mosquit* OR GM arthropod* AND Human Health) OR
(GM mosquit* OR GM arthropod* AND Animal Health) OR
(transgen* mosquit*)

OR

TS= vector* AND arbovir*

OR

TS= Vector* AND Entomopatho* fung*

OR

TS= ((Transmission AND Vector) OR (Epidemiology AND Vector))
AND
TS= (Malaria OR
Dengue OR
Japanese Encephalitis OR
Chikungunya OR
Tick-borne OR
Chagas OR
Filari*)

* TS is for topic.
