# Supplementary material for: Engaging scientists: An online survey exploring the experience of innovative biotechnological approaches to controlling vector-borne diseases
Source: Parasit Vectors. 2015 Aug 10;8:414. doi: 10.1186/s13071-015-0996-x (PMC4530488; doi:10.1186/s13071-015-0996-x)
Supplement: Additional file 3: — Full text of the survey and notes. [file 13071_2015_996_MOESM3_ESM.pdf]

Additional file 3. Invitation and Reminders sent to participate in the web-based survey.

Invitation:

Date: 4 October, 2012 16:09:11 CEST

Subject: Survey Invitation 'Transgenics and Vector-borne Diseases'

Dear colleague,

We are a group of academics from various disciplines ranging from evolutionary biology to anthropology. (See details below) and we would like to invite you to participate in a survey that is part of a research project entitled '**Transgenics and Vector-borne Diseases: Attitudes, Perceptions and Relationships between the scientists and the publics**'.

This project is funded through an exploratory grant by the Institut des SCiences de la Communication from the CNRS, France. [details here : http://www.iscc.cnrs.fr/].

Why do we contact you?

You are the corresponding author of a paper (published after 2005 and referenced in Web of Science databases) dealing with arthropod-borne diseases. Consequently we would value your opinion on transgenic approaches for the control of vector-borne diseases.

What is this study about?

Among the most recent hopes for the control of vector-borne diseases, several methods rely on the use of transgenic insects to interrupt transmission. If the recent technological progress conveys optimism for the viability of these approaches, there is still a widespread appreciation of the complexity of the interactions between science and society.

The intention of this questionnaire is to better understand the link between science, technology and society.

In order to get relevant data, this part of our research is based on a questionnaire. Replying to the questionnaire is not going to be very time-consuming. All information is going to be treated in an anonymous way and personal opinions are not going to be divulgated.

In order to process the results of the questionnaire in the coming months we would appreciate if you could complete this survey as soon as possible after you receive the message and not later than 4 weeks.

Please click on this link to complete the survey:

Start Survey

Thank you very much for your time and support. Feel free to contact the PI if you need additional information.

Christophe Boëte (PI)

Aix-Marseille Université – IRD- EHESS - Unité des Virus Emergents
boete@ird.fr ; cboete@gmail.com

Partners in the project:
Uli Beisel (The Lancaster Environment Centre, UK)
Luisa Reis de Castro (Maastricht University, The Netherlands and Brazil)
Nicolas Césard (Anthropology, MNHN, Paris France)
Guy Reeves (Evolutionary Biology - MPI, Plön Germany)

Start Survey Start Survey

Reminder 1

Date: 22 October, 2012 15:33:00 CEST

Subject: **Vector-borne diseases and Transgenics - A gentle Reminder**

Dear colleague,

We have been inviting you recently to participate in a survey we are conducting dealing with Vector-borne diseases and Trangenics because your email was associated with a paper dealing with arthropods, vectors and/ or vector-borne diseases.

Our project aims at better understanding the links between science, technology and society and it has been funded by the Institut des Sciences de la communication, CNRS (the main government-funded research organization in France). [details here : http://www.iscc.cnrs.fr/].

Whatever your background is (biology, sociology, anthropology...) we would really value your opinion on issues related to vector-borne diseases and the transgenic approaches hence this gentle reminder. **You are legitimate to participate and the first question is going to help you if you have any doubt. Filling the questionnaire should only take 10 to 15 min so we really hope you can join us.**

However if you are not interested you can easily unsubscribe as there is a link to do so in the footer of this message and you won't receive any more reminder later. We are obviously sorry not to get you on board.

If you already started filling it previously you answers have been saved so you won't need much time to complete your answers.

We invite you to click on this link to complete the survey:

Start Survey

Last but not least, all information is going to be treated in an anonymous way and personal opinions are not going to be divulged and we'll keep you informed of the results and outcome of this work.

Thanks again for your time and support and we really appreciate your participation.

Feel free to contact the PI if you need additional information.

Christophe Boëte (PI)

Aix-Marseille Université – IRD- EHESS - Unité des Virus Emergents
boete@ird.fr ; cboete@gmail.com

Partners in the project :

Uli Beisel (The Lancaster Environment Centre, UK)
Luisa Reis de Castro (Maastricht University, The Netherlands and Brazil)
Nicolas Césard (Anthropology, MNHN, Paris France)
Guy Reeves (Evolutionary Biology - MPI, Plön Germany)

Start Survey Start Survey

Reminder 2

Date: 5 November, 2012 20:28:25 CET

Subject: **[Survey Vector-borne diseases & Transgenics] - A gentle Reminder**

Dear colleague,

A couple of weeks ago you have received an invitation to participate in a survey we are conducting dealing with Vector-borne diseases and Trangenics. It is part of a project for which we received financial support by the Institut des Sciences de la communication, CNRS (the main government-funded research organization in France). [details here : http://www.iscc.cnrs.fr/].

You are receiving this reminder today because you have not yet participated. **You do not have to be a specialist of transgenics to participate.**
Maybe you did not have to fill it or maybe you are not interested.

If you are **not interested you can easily unsubscribe** as there is a link to do so in the footer of this message and you won't receive any more reminder later. We are obviously sorry not to get you on board.

If you are interested but if you are not sure that your background and your work fit well with the survey, please go the first question and it should dispel your doubts. Filling the questionnaire should only take 10 to 15 min so we really hope you can join us.

We invite you to click on this link to complete the survey:

Start Survey

Last but not least, all information is going to be treated in an anonymous way and personal opinions are not going to be divulged and we'll keep you informed of the results and outcome of this work.

Thanks again for your time and support and we really appreciate your participation.

Feel free to contact the PI if you need additional information.

Christophe Boëte (PI)

Aix-Marseille Université – IRD- EHESS - Unité des Virus Emergents
boete@ird.fr ; cboete@gmail.com

Partners in the project :
Uli Beisel (The Lancaster Environment Centre, UK)
Luisa Reis de Castro (Maastricht University, The Netherlands and Brazil)
Nicolas Césard (Anthropology, MNHN, Paris France)
Guy Reeves (Evolutionary Biology - MPI, Plön Germany)

Start Survey Start Survey

Reminder 3

Date: 19 November, 2012 14:43:41 CET
Subject: **[Survey] Vector-borne diseases & Transgenics - A gentle Reminder**

Dear colleague,

We are conducting a survey in the area of Vector-borne diseases for which you received an invitation a couple of weeks ago.

We are planning to close the survey soon hence this gentle reminder.

You do not have to be a specialist of transgenics to participate.

Maybe you did not have time to fill it or maybe you are not interested.

If you are **not interested you can easily unsubscribe** as there is a link to do so in the footer of this message and you won't receive any more reminder later. We are obviously sorry not to get you on board.

If you are interested but if you are not sure that your background and your work fit well with the survey, please go the first question and it should dispel your doubts. Filling the questionnaire should only take 10 to 15 min so we really hope you can join us and fill the survey in the coming days.

We invite you to click on this link to complete the survey:

Start Survey

Last but not least, all information is going to be treated in an anonymous way and personal opinions are not going to be divulged and we'll keep you informed of the results and outcome of this work.

Thanks again for your time and support and we really appreciate your participation.

Feel free to contact the PI if you need additional information.

Christophe Boëte (PI)

Aix-Marseille Université – IRD- EHESS - Unité des Virus Emergents
boete@ird.fr ; cboete@gmail.com

Partners in the project :

Uli Beisel (The Lancaster Environment Centre, UK)
Luisa Reis de Castro (Maastricht University, The Netherlands and Brazil)
Nicolas Césard (Anthropology, MNHN, Paris France)
Guy Reeves (Evolutionary Biology - MPI, Plön Germany)

This project is funded by the Institut des Sciences de la communication, CNRS (the main government-funded research organization in France). [details here : http://www.iscc.cnrs.fr/]

Start Survey Start Survey

Reminder 4

Date: 3 December, 2012 18:45:01 CET

Subject: **[Survey Reminder] Vector-borne diseases & Transgenics**

Dear colleague,

We are conducting a survey in the area of Vector-borne diseases for which you received an invitation a couple of weeks ago.

We are planning to close the survey in the coming days hence this gentle reminder.

If you are interested we invite you to click on this link to complete the survey:

Start Survey

If you are not interested we are obviously sorry not to get you on board.

Last but not least, all information is going to be treated in an anonymous way and personal opinions are not going to be divulged and we'll keep you informed of the results and outcome of this work.

Thanks again for your time and support and we really appreciate your participation.

Feel free to contact the PI if you need additional information.

Christophe Boëte (PI)

Aix-Marseille Université – IRD- EHESS - Unité des Virus Emergents
boete@ird.fr ; cboete@gmail.com

Partners in the project :

Uli Beisel (The Lancaster Environment Centre, UK)
Luisa Reis de Castro (Maastricht University, The Netherlands and Brazil)
Nicolas Césard (Anthropology, MNHN, Paris France)
Guy Reeves (Evolutionary Biology - MPI, Plön Germany)

This project is funded by the Institut des Sciences de la communication, CNRS (the main government-funded research organization in France). [details here : http://www.iscc.cnrs.fr/]

Start Survey

Reminder 5

Date: 11 December, 2012 12:38:21 CET

Subject: **[Survey- Last Reminder] Vector-borne diseases & Transgenics**

Dear colleague,

You have received an invitation to participate in a survey we are conducting in the area of Vector-borne diseases but it seems that you have not filled the questionnaire yet.

The survey is still open but **we are going to close it tomorrow on WEDNESDAY, 12th DECEMBER at 11pm GMT hence this gentle reminder.**

If you are interested we invite you to click on this link to complete the survey:

Start Survey

If you are not interested we are obviously sorry not to get you on board.

Thanks again for your time and support and we really appreciate your participation.

Feel free to contact the PI if you need additional information.

Christophe Boëte (PI)

Aix-Marseille Université – IRD- EHESS - Unité des Virus Emergents
boete@ird.fr ; cboete@gmail.com

Partners in the project :

Uli Beisel (The Lancaster Environment Centre, UK)
Luisa Reis de Castro (Maastricht University, The Netherlands and Brazil)
Nicolas César (Anthropology, MNHN, Paris France)
Guy Reeves (Evolutionary Biology - MPI, Plön Germany)

This project is funded by the Institut des Sciences de la communication, CNRS (the main government-funded research organization in France). [details here : http://www.iscc.cnrs.fr/]

Start Survey Start Survey
