# Supplementary figures and images for: Engaging scientists: An online survey exploring the experience of innovative biotechnological approaches to controlling vector-borne diseases
Source: Parasit Vectors. 2015 Aug 10;8:414. doi: 10.1186/s13071-015-0996-x (PMC4530488; doi:10.1186/s13071-015-0996-x)

Fig. S4.

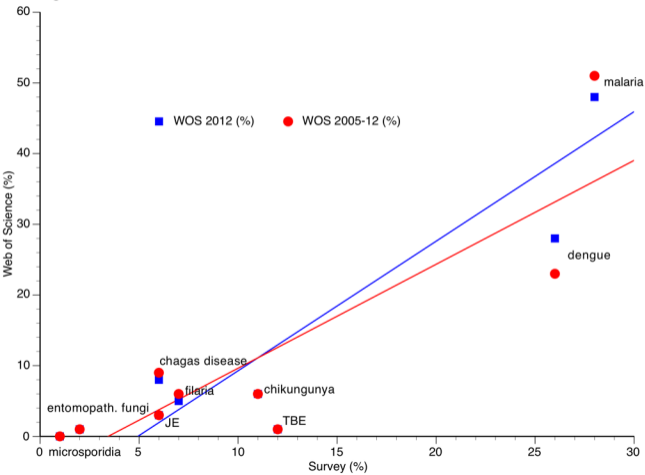

Supplement: Additional file 4: — Representativeness of survey respondents’ field or research (Q2) relative to the publication output of field (based on Web-Of-Science database). [file 13071_2015_996_MOESM4_ESM.pdf]

**Fig. S5.**

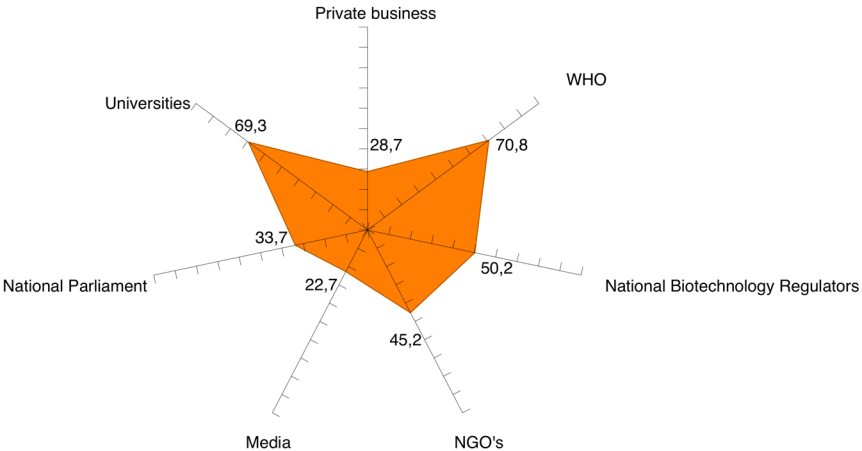

Supplement: Additional file 5: — Level of trust in sources of information about the balance between risks and benefits concerning the release of transgenic mosquitoes (Q25). [file 13071_2015_996_MOESM5_ESM.pdf]

**Fig. S6.**

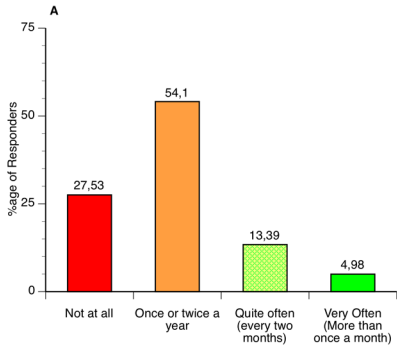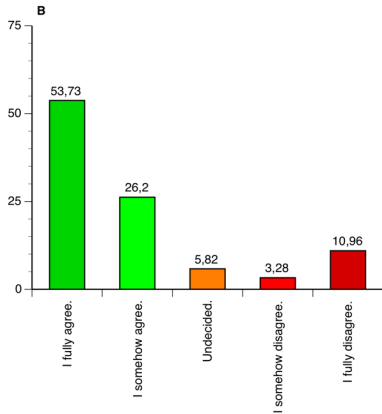

Supplement: Additional file 6: — Reported frequency of communicating and discussing science with a non-specialist audience in events (science fairs, TV/Radio shows, popular press….) not including teaching at a university or at a school (A) (Q17) and its perceived value (B) (Q15). [file 13071_2015_996_MOESM6_ESM.pdf]
