# Supplementary material for: Engaging scientists: An online survey exploring the experience of innovative biotechnological approaches to controlling vector-borne diseases
Source: Parasit Vectors. 2015 Aug 10;8:414. doi: 10.1186/s13071-015-0996-x (PMC4530488; doi:10.1186/s13071-015-0996-x)
Supplement: Additional file 7: — Correspondence analysis between frequency of communication (Q17) and attitudes to early engagement of the public in the research process (Q18). Red crosses represent the level of implication of scientists in communicating and discussing science with a non-specialist audience. Blue squares indicate how early the engagement of the public in the research process is expected by researchers (the lower the number is, the earlier the engagement is). [file 13071_2015_996_MOESM7_ESM.pdf]

Fig. S7.

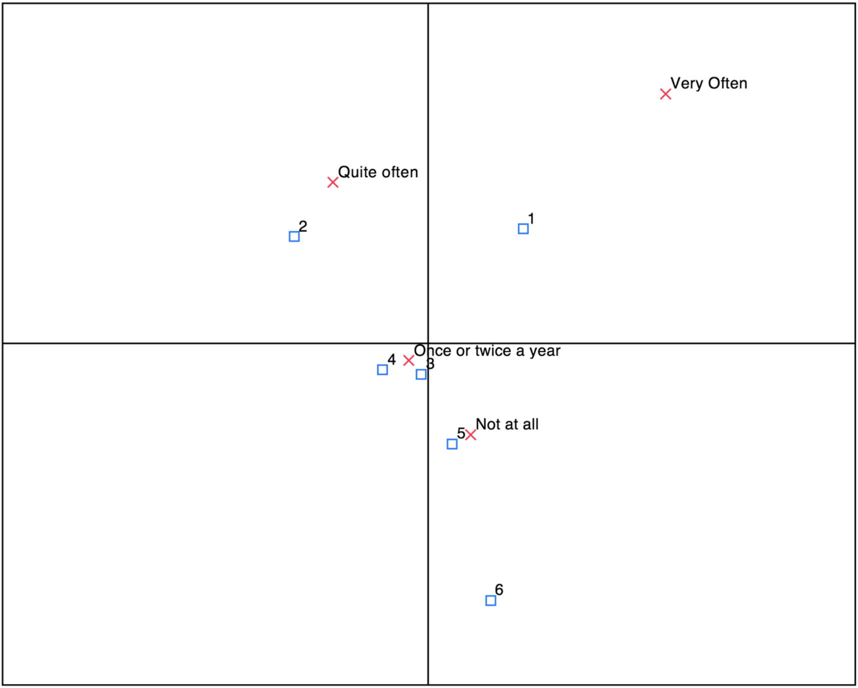

1. Before funding for the project is requested
2. Between the securing of funding and presentation of significant results in scientific journals
3. After or simultaneously with the presentation of significant results in scientific journals
4. Before any permit application for field testing is made
5. After approval for field testing is granted
6. No need to involve the public opinion
